# Music as a Mnemonic to Learn Gesture Sequences in Normal Aging and Alzheimer’s Disease

**DOI:** 10.3389/fnhum.2014.00294

**Published:** 2014-05-12

**Authors:** Aline Moussard, Emmanuel Bigand, Sylvie Belleville, Isabelle Peretz

**Affiliations:** ^1^Rotman Research Institute, Baycrest, University of Toronto, Toronto, ON, Canada; ^2^Laboratoire d’Etude de l’Apprentissage et du Développement (LEAD – CNRS 5022), Université de Bourgogne, Dijon, France; ^3^Centre de Recherche de l’Institut Universitaire de Gériatrie de Montréal (CRIUGM), Université de Montréal, Montréal, QC, Canada; ^4^International Laboratory for Brain, Music and Sound Research (BRAMS), Université de Montréal, Montréal, QC, Canada; ^5^Centre for Research on Brain, Language and Music (CRBLM), McGill University, Montréal, QC, Canada

**Keywords:** music, mnemonic, motor abilities, Alzheimer’s disease, aging, imitation, movement

## Abstract

Strong links between music and motor functions suggest that music could represent an interesting aid for motor learning. The present study aims for the first time to test the potential of music to assist in the learning of sequences of gestures in normal and pathological aging. Participants with mild Alzheimer’s disease (AD) and healthy older adults (controls) learned sequences of meaningless gestures that were either accompanied by music or a metronome. We also manipulated the learning procedure such that participants had to imitate the gestures to-be-memorized in synchrony with the experimenter or after the experimenter during encoding. Results show different patterns of performance for the two groups. Overall, musical accompaniment had no impact on the controls’ performance but improved those of AD participants. Conversely, synchronization of gestures during learning helped controls but seemed to interfere with retention in AD. We discuss these findings regarding their relevance for a better understanding of auditory–motor memory, and we propose recommendations to maximize the mnemonic effect of music for motor sequence learning for dementia care.

## Introduction

Music has been shown to enhance the retention of newly acquired verbal information in normal aging and Alzheimer’s disease (AD). Simmons-Stern et al. (2010) showed that after two exposures, patients with mild AD (mean MMSE = 24/30) were better at recognizing sung than spoken lyrics. Better retention of sung lyrics rather than spoken lyrics was found in a delayed free recall (10 min after learning) in mild AD patients and healthy controls (Moussard et al., 2014). We proposed that dual coding of lyrics and melody lead to a stronger memory trace, which enhances long-term retention. The strong links between music and language suggest a basis for the positive effect of music on verbal memory. According to numerous studies, the shared resources between these domains (Patel, 2008) could explain why adding musical information favors linguistic encoding and memorization. On the other hand, music has been shown to facilitate performance during various kinds of cognitive (including non-linguistic) tasks (Schellenberg, 2005). Accordingly, music may be viewed as having a large positive impact on cognition in general through broad effects on such aspects as attention, emotion, and motivation (arousal effect). The present study further investigates the positive impact of music on memory for non-linguistic material in healthy and AD older adults.

To our knowledge, no study has investigated the effect of a musical accompaniment for motor sequence learning. Yet strong links between music and motor functions suggest that music could represent an interesting aid for motor learning. Long- and short-term musical practice lead to strong plasticity effects in motor brain areas (Wan and Schlaug, 2010; Pantev and Herholz, 2011). Interestingly, listening to music activates motor regions (Brown and Martinez, 2007; Zatorre et al., 2007) and arousing musical pieces can enhance tonus and body posture (Forti et al., 2010). In dementia care, music is used to stimulate movement and alertness in patients with apathy (Cevasco and Grant, 2003; Holmes et al., 2006). A clinical case study showed that two out of three patients tested in the final stage of dementia showed greater reactions to musical than visual or tactile stimulations (Norberg et al., 2003). More generally, many reports from music therapy suggest a strong effect of music on tonus in dementia patients, though further scientific validation of these effects is needed (Aldridge, 1994).

Rhythm seems to be a key component in the relationship between music and motor functions. Motor synchronization is more strongly related to the auditory modality than the visual modality (Repp and Penel, 2004; Patel et al., 2005). In clinical care, auditory–motor synchronization enhances gait and movement production in Parkinson disease (Lim et al., 2005, for a review) and facilitates speech production in aphasia (Racette et al., 2006; Stahl et al., 2011; see Zumbansen et al., 2014, for review). By reinforcing auditory–motor coupling, synchronization during learning could reinforce encoding and facilitate memory retrieval. Moreover, synchronization and auditory–motor coupling could be enhanced by the presence of music accompaniment. In Racette and colleagues’ study, singing (but not speaking) in unison with a demonstrator improves production and retention of sentences in aphasic patients (Racette et al., 2006). Several interpretations can account for a stronger effect of synchronization in musical than non-musical situations. Firstly, music provides cues for rhythmic synchronization and many studies showed strong sensory–motor integration effects, possibly mediated by the mirror neuron system (Chen et al., 2009; D’Ausilio, 2009; Overy and Molnar-Szakacs, 2009). Secondly, the affective component of music and its ability to communicate social and emotional meaning might play a role for synchronization between individuals (Molnar-Szakacs and Overy, 2006; Overy and Molnar-Szakacs, 2009). In the shared affective motion experience (SAME) model, Overy and Molnar-Szakacs (2009) propose that a large network involving the mirror neuron system and the emotional network (anterior insula and limbic system) is involved while experiencing a musical activity, which may explain why music is such a relevant stimulus in all human societies. According to the authors, imitation, synchronization, and shared experience may be key elements for successful therapeutic programs.

In the present pilot study, we investigate the learning and retention of sequences of gestures. We test the influence of two factors, musical accompaniment and synchronization of performance with a demonstrator during encoding, and the interaction between these. Musical accompaniment is expected to enhance recall performance because of links between music processing and motor functions and the general arousing effect of music. However, it could also be that music might distract participants, especially those with AD who may have more attentional deficits (e.g., Gorus et al., 2006). Dual coding resulting from music-based gesture-sequence learning might also lead to a detrimental effect for individuals with fewer cognitive resources. For these reasons, investigating the effects of musical accompaniment on motor memorization will provide both theoretical and clinical insights. Synchronization during the learning of gestures is expected to improve recall performance based on the effect of action on memory, which is well-documented in the literature. For example, learning a list of words describing actions is easier when these actions are mimed during encoding (e.g., Feyereisen, 2009). Additionally, we expected an interaction between synchronization and musical accompaniment, based on the specific links between music and sensory-motor integration described above.

## Materials and Methods

### Participants

Eight AD participants (mean MMSE = 25.2/30; range 23–27) and seven healthy controls participated in the study. Healthy controls were recruited from the participant database of the Research Center of the Geriatric Institute of the University of Montreal (CRIUGM). We recruited AD participants from the Alzheimer Society of Montreal (*N* = 6) and from a cohort of patients followed at the CRIUGM (*N* = 2). AD participants met the NINCDS-ADRDA research criteria for probable AD (McKhann et al., 1984) and the DSM-IV clinical criteria for dementia of the Alzheimer’s type (APA, 1994). Mixed dementias were excluded. Cognitively healthy individuals were used as controls. They were screened for cognitive impairment and selected to match AD patients for age and education. In all participants, exclusion criteria included history of psychiatric or neurological disorders, cerebrovascular diseases, hearing impairment, alcoholism, and dyslexia. All gave informed consent approved by the ethics board of IUGM for their participation in a larger study about music and memory. One control participant was excluded from the analyses because she was unable to complete all conditions, leaving a final pool of eight AD and six controls.

Neuropsychological assessment of participants is presented in Table 1. As expected, AD participants showed lower scores than controls for MMSE (Folstein et al., 1975) and verbal memory for words (Rey’s 15 words; Rey, 1970) and stories (Gély-Nargeot et al., 1997). They also showed slightly inferior scores for verbal comprehension (Token test; De Renzi and Vignolo, 1962). They did not show significant differences from controls for verbal working memory (forward and backward digit spans), auditory attention (TEA, elevator task; Robertson et al., 1994), or praxis (imitation of meaningless gestures). There were no significant differences in the questionnaires of depression (Geriatric Depression Scale; Yesavage et al., 1983) and well-being (Bravo et al., 1996).

**Table 1:** **Neuropsychological assessment**.

	Sex F/M	Age	Education (years)	Cognitive level	Verbal memory (Rev’s 15 words)		Working memory		Attention	Language	Praxis	Mood and well-being	
				MMSE 30	Sum of 5 recalls/75	Recogn. 0.15	Digit span (forward)	Digit span (backward)	TEA (elevator task)/7	Token test/44	Praxis/12	Geriatric depression scale (GDS)/30	Well-being scale 100
**AD**													
HD	F	79	9	23	25	9	5	3	7	40	12	2	80
JO	M	77	17	23	12	2	5	3	6	29	11	0	84
JL	F	67	7	25	32	12	5	5	7	39	12	1	84
AM	F	84	12	25	22	8	7	4	6	32	12	7	54
JE	M	77	7	26	22	9	5	3	7	36	10	17	39
JR	M	79	16	26	17	7	6	4	7	42	12	5	69
RL	M	76	9	27	32	13	5	4	7	37	11	9	64
HU	F	83	16	27	19	4	6	5	7	35	11	6	94
**Average**		**77.8**	**11.6**	25.2***	22.6***	8**	**5.5**	**3.9**	**6.8**	36.3**	**11.4**	**5.9**	**71**
**SD**		**5.2**	**4.2**	**1.6**	**7**	**3.7**	**0.8**	**0.8**	**0.5**	**4.3**	**0.8**	**5.5**	**18.2**
**CONTROLS**													
RJ	F	77	15	28	63	15	5	4	7	44	11	1	85
RD	M	65	11	29	51	15	6	5	7	44	11	3	92
AL	F	70	15	29	62	15	5	4	7	43	12	1	68
LA	M	84	9	29	43	8	7	5	7	42	12	1	87
CB	F	70	8	30	45	12	5	4	7	36	11	4	79
AD	F	82	14	30	61	15	6	4	7	44	12	2	87
**Average**		** 74.7**	**12**	**29.2**	**54.2**	**13.3**	**5.7**	**4.3**	**7**	**42.2**	**11.5**	**2**	**83**
**SD**		**7.5**	**3.1**	**0.8**	**9**	**2.9**	**0.8**	**0.5**	**0**	**3.1**	**0.5**	**1.3**	**8.5**

Asterisks represent differences between AD and controls (**p < 0.05; ***p < 0.01).

Assessment of auditory and musical abilities (Table 2) showed no difference between AD and control participants for musical experience; all were considered non-musicians according to the questionnaire of Ehrlé (1998). There were no differences for auditory perception (repetition of sentences; Moussard et al., 2012), nor musical perception abilities (Scale, Contour/Interval, and Rhythm subtests of the MBEMA; Peretz et al., 2013): all participants showed normal abilities to discriminate changes in melodies, which could either violate the key, the interval size, the contour, or the rhythm. Both groups of participants showed equivalent scores for the recognition of emotions – happiness, sadness, and fear – from short instrumental excerpts (from Vieillard et al., 2008). We also tested participants on their recognition of short instrumental familiar songs (e.g., *Brother John* versus non-familiar lures that were matched in terms of musical characteristics; task from Samson et al., 2012; see also Moussard et al., 2012). Participants showed preserved recognition in their ability to decide whether these songs were familiar or not to them.

**Table 2:** **Auditory and musical assessment**.

	Auditory test	Musical background	Musical abilities (adapted from MBMEA)			Memory for familiar music	Recognition of musical emotions (% correct)		
	Repetition of sentences/24	Questionnaire/27	Scale/20	Interval/contour/20	Rhythm/20	Recognition/8	Joy	Sadness	Fear
**AD**									
HD	23	4	16	15	14	7	–	–	–
JO	21	7	18	17	18	7	81.3	18.8	25
JL	23	4	18	14	16	8	81.3	62.5	56.3
AM	22	6	19	20	18	8	93.8	56.3	43.8
JE	24	3	19	19	19	7	31.3	6.3	31.3
JR	23	9	18	19	19	8	93.8	37.5	43.8
RL	23	3	17	18	17	6	93.8	43.8	25
HU	24	10	19	20	19	8	100	43.8	18.8
**Average**	**22.9**	**5.8**	**18**	**17.8**	**17.5**	**7.4**	**82.1**	**38.4**	**34.8**
**SD**	**1**	**2.7**	**1.1**	**2.3**	**1.8**	**0.7**	**23.5**	**20**	**13.4**
**CONTROLS**									
RJ	24	5	18	16	18	8	100	56.3	25
RD	23	4	20	20	20	8	56.3	31.3	62.5
AL	23	3	18	19	18	5	87.5	50	50
LA	22	3	18	17	18	6	93.8	43.8	68.8
CB	23	4	17	14	15	4	81.3	25	43.8
AD	24	7	17	14	20	8	–	–	–
**Average**	**23.2**	**4.3**	**18**	**16.7**	**18.2**	**6.5**	**83.8**	**41.3**	**50**
**SD**	**0.8**	**1.5**	**1.1**	**2.5**	**1.8**	**1.8**	**16.9**	**13**	**17.1**

### Design

Participants had to learn four different sets of 10 gestures. We first compared two conditions of accompaniment during gesture learning: (1) musical accompaniment, using familiar and danceable folkloric music from Quebec or (2) metronomic accompaniment set to the same tempo as the music of the first condition. Secondly, for each of these, the participant is asked to either (1) observe the gestures to-be-memorized once and then to reproduce them in synchrony with the experimenter before reproducing them alone or (2) observe the gestures twice before reproducing them alone (i.e., experiencing the same amount of exposure to the gestures but without any synchronized production). Thus, each participant learned four sets of gesture sequences: music accompaniment with synchronized production during learning (Music_Sync), music accompaniment without synchrony (Music_NoSync), metronome accompaniment with synchrony (Metronome_Sync), and metronome accompaniment without synchrony (Metronome_NoSync).

### Material

#### Gestures

**Figure 1:**
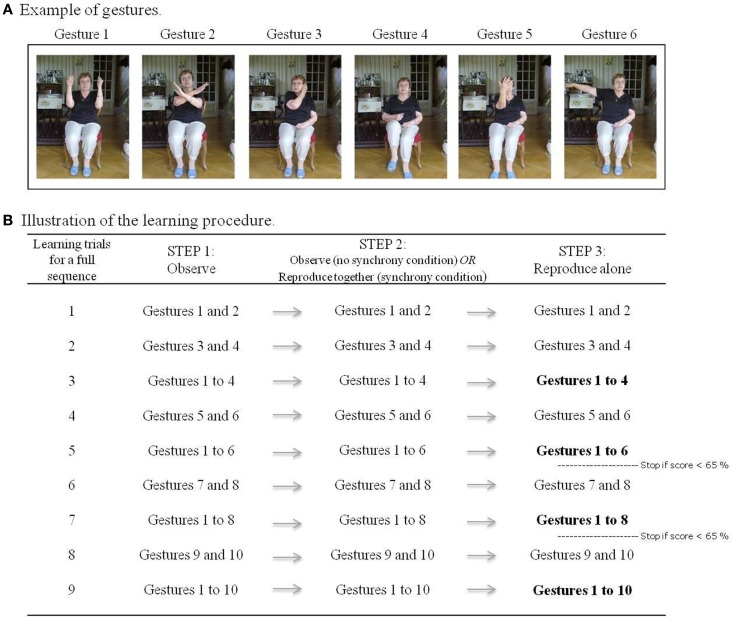
**(A)** Examples of gestures; **(B)** illustration of learning procedure for a full sequence of 10 gestures. Bold font indicates recall trials that are considered in the statistical analysis.

Twelve gestures involving simple and meaningless movements of arms, legs, head, and trunk were selected following the recommendations of a geriatric physiotherapist. They were performed in a secure sitting position (see Figure 1A for an illustration). Four sequences were created with a combination of 10 of the 12 gestures, to-be-learned sequentially. All 12 gestures were performed during a pre-experimental session to ensure that they could easily be done by participants. In case of discomfort with a gesture, it was replaced by one of the two supplementary gestures.

#### Accompaniment

Two musical excerpts were chosen among the repertoire of folkloric music from Quebec (Rigaudon), a style very similar to Irish jigs and reels. They had the same instrumentation, a very similar rhythm and the same tempo (116 bpm, which corresponds to a typical tempo in folkloric Rigaudon music). The excerpts were randomly assigned across the two synchrony conditions (“with” and “without”) for each participant. An audible marker (a “beep” sound) was added to the recording to mark the beginning of each gesture (at the first beat of every measure), making for one gesture approximately every 2 s. To create the metronomic accompaniment, these beep tracks were isolated from the musical tracks, thus keeping the same tempo for the gesture sequences in both musical and metronome conditions. Both metronome tracks were also randomly assigned to the synchrony conditions across participants.

### Procedure

The learning procedure for each gesture-sequence occurred as follows. The participant and experimenter sat face to face. The participant was first familiarized with the complete sequence to-be-memorized by observing the experimenter performing it once, while the corresponding accompaniment played. Then, the first two gestures were taught to the participant following three steps. In the first step, the participant observed the first two gestures performed by the experimenter. The second step varied according to the learning condition, those being either (1) synchrony, where the experimenter’s second performance of each gesture is shadowed by the participant, or (2) without synchrony, where the experimenter’s second performance of gestures is simply observed again by the participant (as in step 1). Finally, in the third step (irrespective of condition), the participant was asked to reproduce the gestures by himself. All learning of subsequent gestures followed the same procedure. After each addition of a pair of gestures, the entire series of learned gestures up until that point was recapitulated. In other words, after adding gestures 3 and 4, gestures 1–4 were performed, and after adding gestures 5 and 6, gestures 1–6 were performed, and so on (always following the steps described above; see Figure 1B for an illustration of the learning procedure). Starting from the recall of the first six gestures, an additional two gestures were only added if participants reached a level of at least 65% during their previous recall (i.e., when they performed the gestures by themselves). This adaptive procedure ensured that the task was suited to each participant’s capabilities and thus was adapted to both groups of participants. The recapitulation of an entire series of learned gestures up until the last pair learned (i.e., gestures 1–4, 1–6, and if performance allowed, 1–8 and 1–10; see in bold in Figure 1B) served as our measure of immediate recall. Immediate recall scores were obtained by adding the scores of these recalls. Participants were tested again 10 min after the end of the learning session for a delayed recall trial: without being exposed to the sequence again, they were asked what they remembered from the sequence of gestures that they have been learned at the beginning of the session.

Music or metronome accompaniment was played for each of the three steps described above during learning and recall. Musical excerpt (see Accompaniment) was always associated to the same sequence of gestures and a given measure or musical phrase was always associated to a specific gesture, whether during observation or reproduction.

The four sequences (the product of the two learning conditions multiplied by the two accompaniment conditions; see Design above) were learned over four different sessions, each a week apart and presented in a randomized order for each subject. Each learning session lasted for an estimated 15 min. All sessions took place in the participant’s home, always at the same hour of the day. Auditory stimuli were presented through a loudspeaker and the entire session was filmed.

### Data analysis

Two judges scored the produced gestures from videos; one of them was not involved in the study (intern students). Judges quantified recall according to four criteria: (1) gestures are present/absent (recalled gestures), (2) gestures are in the right/wrong order in the sequence (order), (3) gestures are well/poorly produced (quality of production), (4) wrong gestures are present (intrusions). When the two judges disagreed (in less than 10% of cases), a third judge (also not involved in the study) scored and arbitrated. Data were then analyzed using non-parametric statistics (Wilcoxon test). There was no clear influence of different conditions on measures of quality of production or intrusions, thus results will focus on the measures of recalled gestures and on gesture order in the sequence.

## Results

Immediate and delayed recall scores highlighted an outlier in the AD group (participant JL), with performance largely higher than the ones from the other seven AD participants. Scores of JL were thus analyzed separately and her data are presented after those of the groups.

### Immediate recall

**Figure 2:**
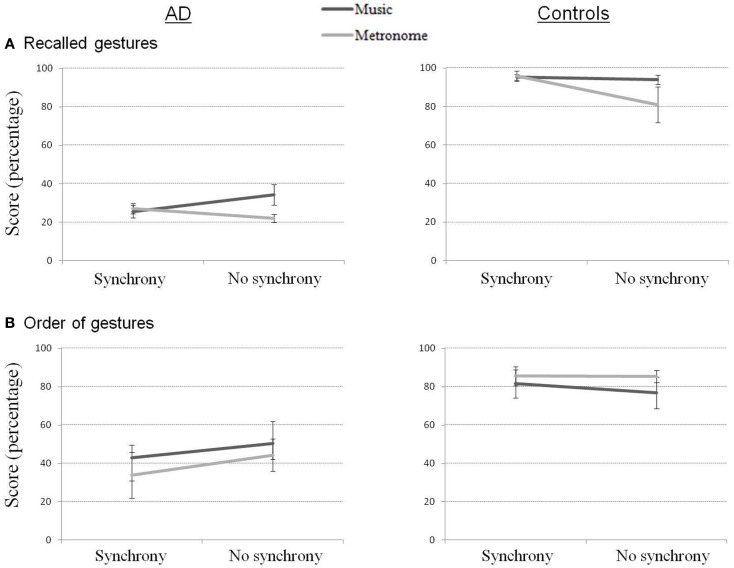
**Recalled gestures (A) and order of gestures (B) in immediate recall for AD and control participants**.

Immediate recall scores for our four measures are presented in Figure 2. Percentage of recalled gestures (out of 10 gestures expected for the complete sequence) showed strong differences between groups for all conditions (all contrasts = *p* < 0.01). Consistent with our hypothesis, performance was worst for both groups in the Metronome_NoSync condition. Statistically though, the only significant contrast was between Metronome_NoSync and Music_NoSync in the AD group (*Z* = 2.11, *p* < 0.05), showing an advantage in the music condition compared to the metronome condition when the gestures were learned without synchrony.

A ratio of well-ordered gestures to total number of gestures produced was derived from the second of our scoring criteria above. This score in AD participants was inferior to that of controls for all conditions (all contrasts = *p* < 0.01) except for the Music_NoSync condition, where the difference between groups did not reach significance. Within each group, no effect of condition reached significance (the only significant difference was the contrast between Music_NoSync and Metronome_Sync in controls).

### Delayed recall

**Figure 3:**
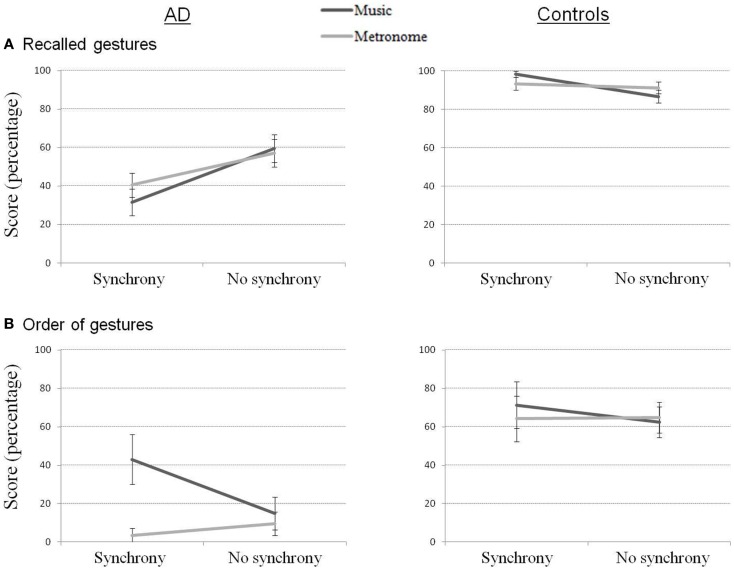
**Recalled gestures (A) and order of gestures (B) in delayed recall for AD and control participants**.

Delayed recall scores (Figure 3) correspond to a ratio of recalled gestures out of the number of gestures that were learned (up to the point of failure). Performance showed again a strong effect of group for all conditions (*p* < 0.05). More correct gestures were recalled with the synchrony conditions for controls (*Z* = 2.03, *p* < 0.05), while more correct gestures were recalled when gestures were learned without synchrony in AD (*Z* = 2.55, *p* < 0.05). When considering the conditions separately, marginal effects confirmed this result. In AD, the Music_NoSync and Metronome_NoSync conditions were better than Music_Sync (*Z* = 1.78, *p* = 0.075 and *Z* = 1.68, *p* = 0.093, respectively) and Metronome_Sync (*Z* = 1.86, *p* = 0.063 and *Z* = 1.83, *p* = 0.068, respectively). In controls, the reverse pattern was shown, with Music_Sync better than Music_NoSync (*Z* = 1.83, *p* = 0.068).

Regarding order of gestures, better scores were observed for controls compared to AD for all conditions (*p* < 0.05) but Music_Sync. The Music_Sync condition was marginally better performed in AD compared to the Metronome_Sync (*Z* = 1.83, *p* = 0.068) and Metronome_NoSync (*Z* = 1.68, *p* = 0.093) conditions.

### Participant JL

Participant JL (MMSE = 25/30), from the AD group, showed relatively well-preserved abilities for the task, especially her score for the number of recalled gestures (similar to controls). Her score did not differ depending on experimental conditions for most of the measures. The only difference was found in immediate recall for gesture order, with worse performance for the music conditions (Music_Sync inferior to Metronome_Sync and Mus_NoSync inferior to Metronome_NoSync, Fisher test, *p* < 0.05).

## Discussion

Music has recently been shown to act as a mnemonic aid for recognition of lyrics in patients with AD (Simmons-Stern et al., 2010, 2012) as well as moderately increasing the retention of lyrics (Moussard et al., 2012, 2014). Music has also been shown to be strongly linked to motor functions (e.g., Zatorre et al., 2007). The present pilot study is the first study to investigate the potential of music as an aid for learning and retention of non-verbal information such as a sequence of gestures in both healthy old adults and AD individuals. The performance of participants was measured in terms of the number of gestures recalled and their order in the sequence of gestures, in both immediate and 10-min delayed recalls. Results showed different patterns for each group. AD participants showed a modest advantage for the music condition, as seen in the significantly greater percentage of gestures recalled in immediate recall and the marginal effect regarding the order of gestures in the sequence in delayed recall. AD participants also showed better performance for the sequences that were learned without synchrony (compared to with synchrony) in delayed recall. Control participants did not show any clear influences of accompaniment (music or metronome) but did show better scores in delayed recall when gestures were learned in synchrony.

Effects of synchronized production of gestures during learning were shown in delayed recall, with synchrony being helpful for controls but detrimental to AD participants. For controls, being more physically active during learning may help with maintaining motivation and attention. Dual or embodied coding of gestures might also have reinforced the memory trace, benefiting from tight auditory–motor coupling in the brain (Zatorre et al., 2007). This multimodal coding leads to multiple pathways involving a more complex brain network and deeper encoding (Craik and Tulving, 1975; Brown and Palmer, 2012). Moreover, the superiority of synchrony learning in the controls seems to be mainly driven by the fact that the Music_Sync condition led to the best performance out of all conditions. This finding supports our hypothesis that there is a positive interaction between music and motor synchrony during learning (Racette et al., 2006). The rhythmical and/or the emotional and affective richness of musical stimuli (see SAME model, Overy and Molnar-Szakacs, 2009) may have facilitated synchronization during learning and in turn strengthened the learning process. In AD participants, it may have been that imitating the gestures in synchrony was more demanding due to a limited ability to maintain attention or the fact that participants did not put as much effort into encoding when additional cues were provided (compared to the condition without this support). It is also possible that AD participants were not able to benefit from the synchrony learning to the same extent as the controls because of potential difficulties in motor functions due to the disease (e.g., Kurlan et al., 2000).

The fact that AD participants showed a modest increase in performance in the music condition for some of the measures while controls (and JL, the best performer of AD group) did not seem to be influenced by the accompaniment (either music or metronome) suggests that music is of greater benefit to those with more pronounced cognitive impairment. This might be due to several factors. Firstly, the dual coding between motor and auditory information may have reinforced the memory trace, ensuring higher quality of encoding and recall. It is important to note that adding musical information does not overload participant’s attention as we might have expected considering limited cognitive resources. Secondly, the arousing characteristic of music is known to enhance short-term cognitive efficiency (e.g., Schellenberg et al., 2007). In a case study by Johnson et al. (1998), an AD patient showed improved performance at a spatial–temporal task after listening to an arousing musical excerpt. Enjoyable and energetic music could put participants into a more alert state, as well as decrease any stress related to the test situation and, in turn, help compensate for the cognitive impairment.

The positive effect of music compared to metronome for AD participants was smaller than anticipated. It is possible that the metronome condition played a mnemonic role itself and helped the learning, more so than learning in silence. Research in Parkinson’s disease has shown that a regular beat was most often as helpful as music to support motor functions such as gait (Thaut et al., 2001). The imposed tempo for learning and retrieval may have helped structure the sequence during encoding and/or assist in planning the motor actions, making recall more automatic. This would have to be confirmed in a further study with a silent control condition.

It may also be the case that the associations between gestures and music were not optimal. Rigaudon is a style of music similar to Irish jigs, where the same musical phrase is repeated and only slightly modified throughout the excerpt. While this music is appropriate because it is familiar to older adults and stimulates movement, it may be better to use music with more variety. This could assist in the memorization of gestures as it would provide more distinctive cues and anchor points to associate with them. Similarly, using musical rhythm and its variability instead of having gestures on regular beats only could also provide more cues and help structure the sequence into smaller units (chunks; see Purnell-Webb and Speelman, 2008).

To conclude, music might be used as a mnemonic for gesture-sequence learning in AD patients, although synchronization of gesture production during encoding does not help performance. In healthy matched controls, synchronization during learning enhanced retention and interacted positively with music, thus supporting models of auditory–motor integration in healthy individuals. The main limitation of the study concerns the small sample size. With larger groups of participants, further studies will allow generalization of results to the AD population. Moreover, larger samples would allow correlational analyses aiming to determine profiles of individuals who would most benefit from music and/or synchronization for gesture-sequence learning. Further studies are also necessary to try to maximize the effect provided by the musical accompaniment and to assess how the to-be-learned gestures can be linked to the everyday needs of patients. For example, our procedure could be used to teach patients the series of gestures needed to use their new coffee machine or DVD player, to warm frozen food in the microwave, or to start load of laundry.

## Conflict of Interest Statement

The authors declare that the research was conducted in the absence of any commercial or financial relationships that could be construed as a potential conflict of interest.

## References

[B1] Aldridge D. (1994). Alzheimer’s disease: rhythm, timing and music as therapy. Biomed. Pharmacother. 48, 275–281 10.1016/0753-3322(94)90172-4 7858157

[B2] APA. (1994). Diagnostic and Statistical Manual of Mental Disorders, 4th Edn Washington, DC: American Psychiatric Association

[B3] Bravo G. Gaulin P. Dubois M.-F. (1996). Validation d’une échelle de bien-être général auprès d’une population francophone âgée de 50 à 75 ans. Can. J. Aging 15, 112–128 10.1017/S0714980800013325

[B4] Brown R. M. Palmer C. (2012). Auditory-motor learning influences auditory memory for music. Mem. Cognit. 40, 567–578 10.3758/s13421-011-0177-x 22271265

[B5] Brown S. Martinez M. J. (2007). Activation of premotor vocal areas during musical discrimination. Brain Cogn. 63, 59–69 10.1016/j.bandc.2006.08.006 17027134

[B6] Cevasco A. M. Grant R. E. (2003). Comparison of different methods for eliciting exercise-to-music for clients with Alzheimer’s disease. J. Music Ther. 40, 41–56 10.1093/jmt/40.1.41 17590967

[B7] Chen J. L. Penhune V. B. Zatorre R. J. (2009). The role of auditory and premotor cortex in sensorimotor transformations. Ann. N. Y. Acad. Sci. 1169, 15–34 10.1111/j.1749-6632.2009.04556.x 19673752

[B8] Craik F. I. M. Tulving E. (1975). Depth of processing and the retention of words in episodic memory. J. Exp. Psychol. Gen. 104, 268–294 10.1037/0096-3445.104.3.26824156261

[B9] D’Ausilio A. (2009). Mirror-like mechanisms and music. ScientificWorldJournal 16, 1415–1422 10.1100/tsw.2009.160 20024515 PMC5823102

[B10] De Renzi E. Vignolo L. A. (1962). The token test: a sensitive test to detect receptive disturbances in aphasics. Brain 85, 665–678 10.1093/brain/85.4.665 14026018

[B11] Ehrlé N. (1998). Traitement Temporel de L’information Auditive et Lobe Temporal. Unpublished Doctoral Dissertation, Université de Reims, Reims.

[B12] Feyereisen P. (2009). Enactment effects and integration processes in younger and older adults’ memory for actions. Memory 17, 374–385 10.1080/09658210902731851 19221926

[B13] Folstein M. F. Folstein S. E. McHugh P. R. (1975). Mini mental state: a practical method for grading the cognitive state of patients for the clinician. J. Psychiatr. Res. 12, 189–198 10.1016/0022-3956(75)90026-6 1202204

[B14] Forti S. Filipponi E. Di Berardino F. Barozzi S. Cesarani A. (2010). The influence of music on static posturography. J. Vestib. Res. 20, 351–356 10.3233/VES-2010-0361 20826933

[B15] Gély-Nargeot M. C. Cadilhac C. Touchon J. Nespoulous J. L. (1997). “La mémoire de textes chez les sujets sains et déments: application d’un nouvel outil d’évaluation pour neuropsychologues: mémo-textes,” in Perception Auditive et Compréhension du Langage, eds Lambert J. Nespoulous J. L. (Marseille: Solal), 273–293

[B16] Gorus E. De Raedt R. Lambert M. Lemper J. C. Mets T. (2006). Attentional processes discriminate between patients with mild Alzheimer’s disease and cognitively healthy elderly. Int. Psychogeriatr. 18, 539–549 10.1017/s1041610205002723 16472408

[B17] Holmes C. Knights A. Dean C. Hodkinson S. Hopkins V. (2006). Keep music live: music and the alleviation of apathy in dementia subjects. Int. Psychogeriatr. 18, 623–630 10.1017/S1041610206003887 16805928

[B18] Johnson J. K. Cotman C. W. Tasaki C. S. Shaw G. L. (1998). Enhancement of spatial-temporal reasoning after a Mozart listening condition in Alzheimer’s disease: a case study. Neurol. Res. 20, 666–672 9864729 10.1080/01616412.1998.11740582

[B19] Kurlan R. Richard I. H. Papka M. Marshall F. (2000). Movement disorders in Alzheimer’s disease: more rigidity of definitions is needed. Mov. Disord. 15, 24–29 10.1002/1531-8257(200001)15:1<24::AID-MDS1006>3.0.CO;2-X 10634238

[B20] Lim I. Van Wegen E. De Goede C. Deutekom M. Nieuwboer A. Willems S. (2005). Effects of external rhythmical cueing on gait in patients with Parkinson’s disease: a systematic review. Clin. Rehabil. 19, 695–713 10.1191/0269215505cr906oa 16250189

[B21] McKhann G. Drachman D. Folstein M. Katzman R. Proce D. Stadlan E. M. (1984). Clinical diagnosis of Alzheimer disease: report of the NINCDS-ADRDA work group under the auspices of Health and human services task force on Alzheimer’s disease. Neurology 34, 939–944 10.1212/WNL.34.7.939 6610841

[B22] Molnar-Szakacs I. Overy K. (2006). Music and mirror neurons: from motion to ‘e’motion. Soc. Cogn. Affect. Neurosci. 1, 235–241 10.1093/scan/nsl029 18985111 PMC2555420

[B23] Moussard A. Bigand E. Belleville S. Peretz I. (2014). Learning sung lyrics aids retention in normal aging and Alzheimer’s disease. Neuropsychol. Rehabil. (in press). 10.1080/09602011.2014.917982 24881953

[B24] Moussard A. Bigand E. Belleville S. Peretz I. (2012). Music as an aid to learn new verbal information in Alzheimer’s disease. Music Percept. 29, 521–531 10.1525/mp.2012.29.5.521

[B25] Norberg A. Melin E. Asplund K. (2003). Reactions to music, touch and object presentation in the final stage of dementia: an exploratory study. Int. J. Nurs. Stud. 40, 473–479 10.1016/S0020-7489(03)00062-2 3536774

[B26] Overy K. Molnar-Szakacs I. (2009). Being together in time: musical experience and the mirror neuron system. Music Percept. 26, 486–504 10.1525/MP.2009.26.5.489

[B27] Pantev C. Herholz S. C. (2011). Plasticity of the human auditory cortex related to musical training. Neurosci. Biobehav. Rev. 35, 2140–2154 10.1016/j.neubiorev.2011.06.010 21763342

[B28] Patel A. D. (2008). Music, Language, and the Brain. New York: Oxford University Press

[B29] Patel A. D. Iversen J. R. Chen Y. Repp B. H. (2005). The influence of metricality and modality on synchronization with a beat. Exp. Brain Res. 163, 226–238 10.1007/s00221-004-2159-8 15654589

[B30] Peretz I. Gosselin N. Nan Y. Caron-Caplette E. Trehub S. Béland R. (2013). A novel tool for evaluating children’s musical abilities across age and culture. Front. Syst. Neurosci. 7:30 10.3389/fnsys.2013.00030 23847479 PMC3707384

[B31] Purnell-Webb P. Speelman C. P. (2008). Effects of music on memory for text. Percept. Mot. Skills 106, 927–957 10.2466/pms.106.3.927-957 18712216

[B32] Racette A. Bard C. Peretz I. (2006). Making non-fluent aphasics speak: sing along! Brain 129, 2571–2584 10.1093/brain/awl250 16959816

[B33] Repp B. H. Penel A. (2004). Rhythmic movement is attracted more strongly to auditory than to visual rhythms. Psychol. Res. 68, 252–270 10.1007/s00426-003-0143-8 12955504

[B34] Rey A. (1970). L’examen Clinique en Psychologie. Paris: PUF

[B35] Robertson I. H. Ward A. Ridgeway V. Nimmo-Smith I. N. (1994). Test of Everyday Attention. Bury St Edmunds: Thames Valley Test

[B36] Samson S. Baird A. Moussard A. Clément S. (2012). Does pathological aging affect musical learning and memory? Music Percept. 29, 493–500 10.1525/mp.2012.29.5.493

[B37] Schellenberg E. G. (2005). Music and cognitive abilities. Curr. Dir. Psychol. Sci. 14, 317–320 10.1111/j.0963-7214.2005.00389.x

[B38] Schellenberg E. G. Nakata T. Hunter P. G. Tamoto S. (2007). Exposure to music and cognitive performance: tests of children and adults. Psychol. Music 35, 5–19 10.1177/0305735607068885

[B39] Simmons-Stern N. R. Budson A. E. Ally B. A. (2010). Music as a memory enhancer in patients with Alzheimer’s disease. Neuropsychologia 48, 3164–3167 10.1016/j.neuropsychologia.2010.04.033 20452365 PMC2914108

[B40] Simmons-Stern N. R. Deason R. G. Brandler B. J. Frustace B. S. O’Connor M. K. Ally B. A. (2012). Music-based memory enhancement in Alzheimer’s disease: promise and limitations. Neuropsychologia 50, 3295–3303 10.1016/j.neuropsychologia.2012.09.019 23000133 PMC3567773

[B41] Stahl B. Kotz S. A. Henseler I. Turner R. Geyer S. (2011). Rhythm in disguise: why singing may not hold the key to recovery from aphasia. Brain 134(Pt 10), 3083–3093 10.1093/brain/awr240 21948939 PMC3187543

[B42] Thaut M. H. McIntosh K. W. McIntosh G. C. Hoemberg V. (2001). Auditory rhythmicity enhances movement and speech motor control in patients with Parkinson’s disease. Funct. Neurol. 16, 163–172 11495422

[B43] Vieillard S. Peretz I. Gosselin N. Khalfa S. Gagnon L. Bouchard B. (2008). Happy, sad, scary and peaceful musical excerpts for research on emotions. Cogn. Emot. 22, 720–752 10.1080/02699930701503567

[B44] Wan C. Y. Schlaug G. (2010). Music making as a tool for promoting brain plasticity across the life span. Neuroscientist 16, 566–577 10.1177/1073858410377805 20889966 PMC2996135

[B45] Yesavage J. A. Brink T. L. Rose T. L. Lum O. Huang V. Adey M. B. (1983). Development and validation of a geriatric depression screening scale: a preliminary report. J. Psychiatr. Res. 17, 37–49 10.1016/0022-3956(82)90033-4 7183759

[B46] Zatorre R. J. Chen J. L. Penhune V. B. (2007). When the brain plays music: auditory-motor interactions in music perception and production. Nat. Rev. Neurosci. 8, 547–558 10.1038/nrn2152 17585307

[B47] Zumbansen A. Peretz I. Hébert S. (2014). Melodic intonation therapy: back to basics for future research. Front. Neurol. 5:7 10.3389/fneur.2014.00007 24478754 PMC3904283

